# Metabolome-Wide Association Study of Neovascular Age-Related Macular Degeneration

**DOI:** 10.1371/journal.pone.0072737

**Published:** 2013-08-27

**Authors:** Melissa P. Osborn, Youngja Park, Megan B. Parks, L. Goodwin Burgess, Karan Uppal, Kichun Lee, Dean P. Jones, Milam A. Brantley

**Affiliations:** 1 Vanderbilt Eye Institute, Vanderbilt University Medical Center, Nashville, Tennessee, United States of America; 2 Department of Medicine, Emory University School of Medicine, Atlanta, Georgia, United States of America; 3 Department of Industrial Engineering, Hanyang University, Seoul, Korea; National Eye Institute, United States of America

## Abstract

**Purpose:**

To determine if plasma metabolic profiles can detect differences between patients with neovascular age-related macular degeneration (NVAMD) and similarly-aged controls.

**Methods:**

Metabolomic analysis using liquid chromatography with Fourier-transform mass spectrometry (LC-FTMS) was performed on plasma samples from 26 NVAMD patients and 19 controls. Data were collected from mass/charge ratio (*m/z*) 85 to 850 on a Thermo LTQ-FT mass spectrometer, and metabolic features were extracted using an adaptive processing software package. Both non-transformed and log2 transformed data were corrected using Benjamini and Hochberg False Discovery Rate (FDR) to account for multiple testing. Orthogonal Partial Least Squares-Discriminant Analysis was performed to determine metabolic features that distinguished NVAMD patients from controls. Individual *m/z* features were matched to the Kyoto Encyclopedia of Genes and Genomes database and the Metlin metabolomics database, and metabolic pathways associated with NVAMD were identified using MetScape.

**Results:**

Of the 1680 total *m/z* features detected by LC-FTMS, 94 unique *m/z* features were significantly different between NVAMD patients and controls using FDR (q = 0.05). A comparison of these features to those found with log2 transformed data (n = 132, q = 0.2) revealed 40 features in common, reaffirming the involvement of certain metabolites. Such metabolites included di- and tripeptides, covalently modified amino acids, bile acids, and vitamin D-related metabolites. Correlation analysis revealed associations among certain significant features, and pathway analysis demonstrated broader changes in tyrosine metabolism, sulfur amino acid metabolism, and amino acids related to urea metabolism.

**Conclusions:**

These data suggest that metabolomic analysis can identify a panel of individual metabolites that differ between NVAMD cases and controls. Pathway analysis can assess the involvement of certain metabolic pathways, such as tyrosine and urea metabolism, and can provide further insight into the pathophysiology of AMD.

## Introduction

Age-related macular degeneration (AMD) remains a leading cause of irreversible vision loss in older individuals in developed countries. Approximately 1.75 million people in the United States over the age of forty suffer from advanced stages of the disease, and this number is projected to approach 3 million by 2020 [1]. Neovascular AMD (NVAMD), in which blood or serous fluid leaks from abnormal choroidal or retinal vessels, is responsible for the majority of AMD-related vision loss [2].

Risk of developing this complex disease is influenced by genetic, demographic, and environmental factors. Genetic variants in the complement factor H gene (*CFH*) and the age-related maculopathy susceptibility 2/HtrA serine peptidase 1 (*ARMS2/HTRA1*) locus have been strongly and consistently associated with AMD. Polymorphisms in the genes coding for complement components 2 and 3 (*C2*, *C3*) and complement factors B and I (*CFB*, *CI*) have also been linked to AMD [3].

In addition to genetic factors, demographic and environmental variables such as older age, smoking, and light exposure influence risk of developing AMD [4]. Single biomarkers, such as homocysteine and carboxyethylpyrrole, have been used to approximate the biochemical microenvironment characterizing AMD with varying success [5], [6]. The limitations of such single biomarker studies could reflect heterogeneity of disease among patients or complexity of metabolite interactions among multiple pathways.

High-resolution metabolic profiling with liquid chromatography-mass spectrometry (LC-MS) can be used to comprehensively evaluate up to 7000 metabolites in plasma and has the potential to identify specific collections of metabolites that are altered in AMD [7], [8]. Metabolomic analysis of serum and plasma has revealed panels of metabolites that distinguish patients with cardiovascular disease [9], breast cancer [10], [11], Parkinson’s disease [12], and diabetes [13]. Importantly, this technique is able to differentiate between individuals despite intra-individual variation due to factors such as dietary intake [14]. As evidence supports the systemic nature of AMD [15], plasma metabolic profiling could reveal clinically-relevant biomarkers that are indicative of AMD development or progression prior to clinical manifestation.

The purpose of this study was twofold: first, to produce quantitative data evaluating environmental markers associated with NVAMD; second, to determine whether plasma metabolic profiling can detect metabolic differences between NVAMD patients and controls in a discovery cohort. The results of this work may lead to a better understanding of AMD pathophysiology and to biomarker discovery.

## Methods

### Ethics Statement

This case-control study was approved by the Vanderbilt University Human Research Protection Program. Research adhered to the tenets of the Declaration of Helsinki and was conducted in accordance with Health Insurance Portability and Accountability Act regulations. Written informed consent was obtained from all participants prior to study enrollment.

### Study Participants

Individuals over the age of 60 were recruited from the Retina Division at the Vanderbilt Eye Institute. Cases were diagnosed with NVAMD in one or both eyes, and controls had no clinical signs of AMD. Exclusion criteria included active uveitis or ocular infection, the presence of any retinopathy other than AMD, and any ocular surgery within the 60 days prior to enrollment. Patients with diabetes mellitus were excluded due to the effects of hyperglycemia on metabolic function [16]. Disease status was confirmed by high-resolution fundus photography. Fifty-degree fundus images were examined by a masked retina specialist for the presence of the following AMD-related findings: neurosensory retinal detachment, pigment epithelial detachment, sub- and/or intra-retinal exudation (hemorrhage and/or lipid), choroidal neovascularization, and fibrovascular tissue. Smoking history and dietary supplement history were obtained from all participants.

### Sample Collection

At the time of study enrollment, blood was drawn from study participants using a 23-gauge butterfly needle. Approximately 8 mL blood was immediately transferred to two 4 mL blood collection tubes containing 7.2 mg K_2_ EDTA each. These tubes were centrifuged at 4°C to remove blood cells, and 2 mL supernatant from each tube was transferred to one of two 15 mL conical tubes. Plasma was immediately frozen at −80°C and not thawed prior to analysis.

### Metabolomic Analysis

Frozen plasma samples from 45 individuals (26 NVAMD patients and 19 controls) were thawed and analyzed by liquid chromatography with Fourier-transform mass spectrometry (LC-FTMS) at Emory University as previously described [14]. Briefly, 100 µL plasma sample aliquots were treated with acetonitrile, spiked with internal standard mix, and centrifuged at 13,000×g for 2 minutes to remove protein prior to being loaded onto a Shimadzu autosampler. Anion exchange columns were equilibrated to the initial condition for 2 minutes prior to the next sample injection. Samples were fractionated with a formate gradient, ionized with electrospray ionization in the positive mode, and detected with an LTQ-FT spectrometer (Thermo, San Jose, CA) from mass/charge ratio (*m/z*) 85 to 850 over 10 minutes. Peak extraction and quantification of ion intensities were performed by an adaptive processing software package (apLCMS) [17], which provided tables containing *m/z* values, retention time, and integrated ion intensity for each *m/z* feature.

### Data Analysis

Descriptive statistics for all demographic and clinical variables were calculated. Comparisons between cases and controls were made using the two-tailed t-test for continuous data (e.g., age) and the two-tailed Fisher exact test for categorical data (e.g., gender, race, smoking, and presence of comorbid conditions). Differences in *m/z* features between cases and controls were determined using Benjamini and Hochberg False Discovery Rate (FDR) with q = 0.05 to account for multiple testing. Bioinformatic analyses included principal component analysis (PCA) and orthogonal partial least squares-discriminatory analysis (OPLS-DA), using Pirouette version 4.0 (InfoMetrix) as complementary approaches to identify metabolic features that distinguish AMD patients from controls. Pearson correlations were determined for *m/z* features and were evaluated on a targeted basis for discriminatory features. Support Vector Machine (SVM) analysis was performed using the svm() function in the R package e1071, which provides an interface to the libSVM library, [18] http://www.csie.ntu.edu.tw/~cjlin/libsvm. Default settings were used to train and test the models based on classification with the linear, polynomial, and Gaussian kernels. The performance of the models was evaluated using the 10-fold and leave-one-out cross-validation methods.

### Metabolite Annotation and Pathway Analysis

Discriminatory *m/*z features were annotated on a targeted basis using Metlin (http://metlin.scripps.edu/) and Kyoto Encyclopedia of Genes and Genomes (KEGG; http://www.genome.jp/kegg/) databases. As indicated by instrument specifications and documented mass resolutions and accuracies, the mass accuracy used should be within 5 ppm, which is sufficient to predict the elemental composition for many low molecular weight metabolites. Parameter settings optimized for moderately high-throughput analysis of plasma on the LTQ-FT resulted in observed Δ ppm for measured versus absolute *m/z* of up to 8 ppm for known standards [14]; consequently, database searches were done with 10 ppm tolerance. Previous comparisons showed that searches with 10 ppm increased the number of matches by <10% compared to a 5 ppm tolerance [19], indicating that use of this search parameter to avoid missing correct matches does not substantially increase the number of incorrect matches. For many *m/z*, multiple matches were present. In some cases, such multiple matches were easy to address because they included multiple database entries for the same chemical or a range of non-physiologic isomers of a common metabolite. In previous studies using coelution with authentic standards and in ion dissociation (MS/MS) studies, we have found matches to known metabolic intermediates to be correct 60–80% of the time [7], [14], [19], [20], [21]. Analysis of a set of randomly generated high-resolution *m/z* values showed that 14% matched metabolites in Metlin. Correlation analyses described below were used to address this limitation. In some cases, large numbers of phytochemicals or large numbers of bile acids share elemental compositions. For these, we would simply summarize as “phytochemicals” or “bile acids.” Confirmed identifications are given when available. Characterization of unidentified metabolites remains a challenge due to the large number of features, relatively low abundances, and the lack of commercial sources for authentic standards, but the accurate mass and retention times obtained should enable identification of these metabolites in the future.

We used correlation analyses to improve confidence in interpretation. The correlation analyses were summarized in a spreadsheet of Pearson correlation coefficients for every *m/z* feature with every other *m/z* feature across all samples. This table allowed selection of the *m/z* features in rank order of Pearson correlation with each *m/z* of interest. Groups of *m/z* features highly correlated with the feature of interest were then searched as a batch in Metlin. These searches provided matches for multiple related chemicals, such as different tripeptides containing similar amino acids, while corresponding searches of randomly selected *m/z* features provided few matches. Formal testing was not attempted because over half of the *m/z* detected do not match metabolites in the databases, indicating that the currently defined metabolic pathways are incomplete. Additional pathway analyses with Metscape were used to provide complementary information on possible metabolic differences between NVAMD and controls.

### Data Access

Data files relevant to this publication will be made available for non-profit use by researchers upon request.

## Results

### Subject Characteristics

The study population consisted of 45 subjects, including 26 patients with NVAMD in one or both eyes and 19 controls without signs of AMD. The mean age of NVAMD patients (76.0±5.7 years) did not differ from that of controls (76.4±4.8 years; p = 0.79). The percentages of males and females did not significantly differ between the groups (p = 0.07), and all participants were Caucasian.

Surveyed environmental factors did not differ between NVAMD and control groups. Each group had two cigarette smokers (p = 1.0). Twenty-three (88.5%) of the NVAMD patients took vitamin supplements, compared to 63.2% of the control patients (p = 0.07). Of the 23 NVAMD patients taking oral vitamin supplements, 15 were taking eye vitamins. Twelve of the 26 NVAMD patients (46.1%) had active choroidal neovascularization at the time of the blood draw, and 9 (34.6%) patients had received an intravitreal injection of anti-vascular endothelial growth factor (VEGF) within the preceding two months.

Comorbid conditions are common in this age group, and an imbalance in comorbidities between the NVAMD and control groups could confound the results of the study. Specifically, coronary artery disease and associated conditions have been linked to AMD [22]. Therefore, we examined the presence of comorbidities in the study population. There was no significant difference in the percentage of patients in each group with the following: coronary artery disease (NVAMD 27%, control 16%, p = 0.481), hypertension (NVAMD 65%, control 58%, p = 0.757), hyperlipidemia (NVAMD 35%, control 32%, p = 1.00), diabetes (NVAMD 0%, control 0%, p = 1.00), or history of cancer (NVAMD 12%, control 3%, p = 0.627).

### High-resolution Mass Spectral Data

**Figure 1:**
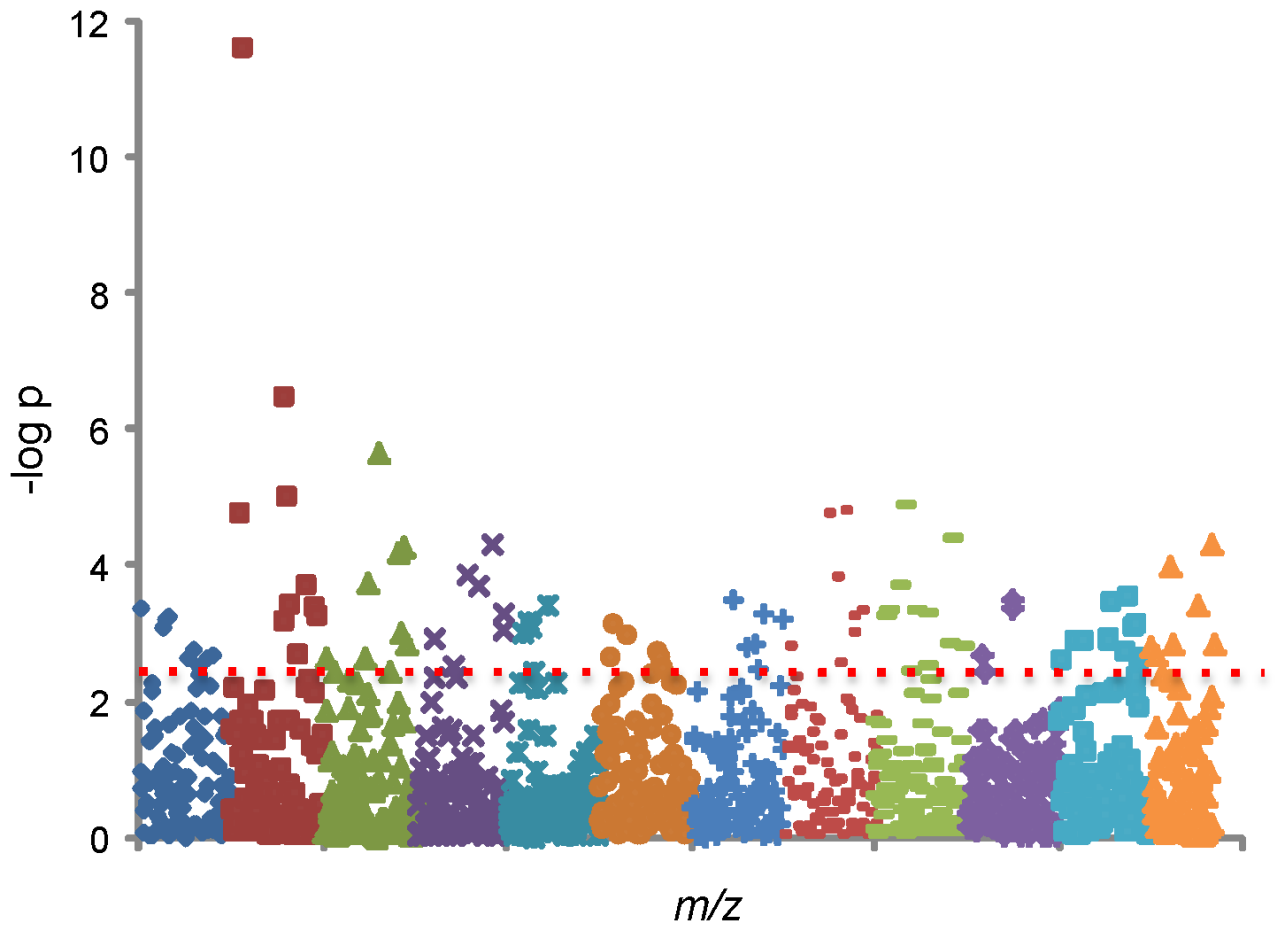
Metabolome-wide association study of NVAMD. This Manhattan plot depicts FDR analysis of 1168 *m/z* features comparing 26 NVAMD patients and 19 controls. The negative logp value is plotted against the *m/z* features. The x-axis represents the m/z of the features, ordered in increasing value from left (85) to right (850). The coloring of the symbols is arbitrary. A total of 94 features significantly differed between NVAMD and controls at an FDR of q = 0.05 (above horizontal dashed line). Two of the features shared the same *m/z* value but had different retention times, so the 95 features shown in this figure were collapsed to 94 in the search of *m/z* in the databases.

**Figure 2:**
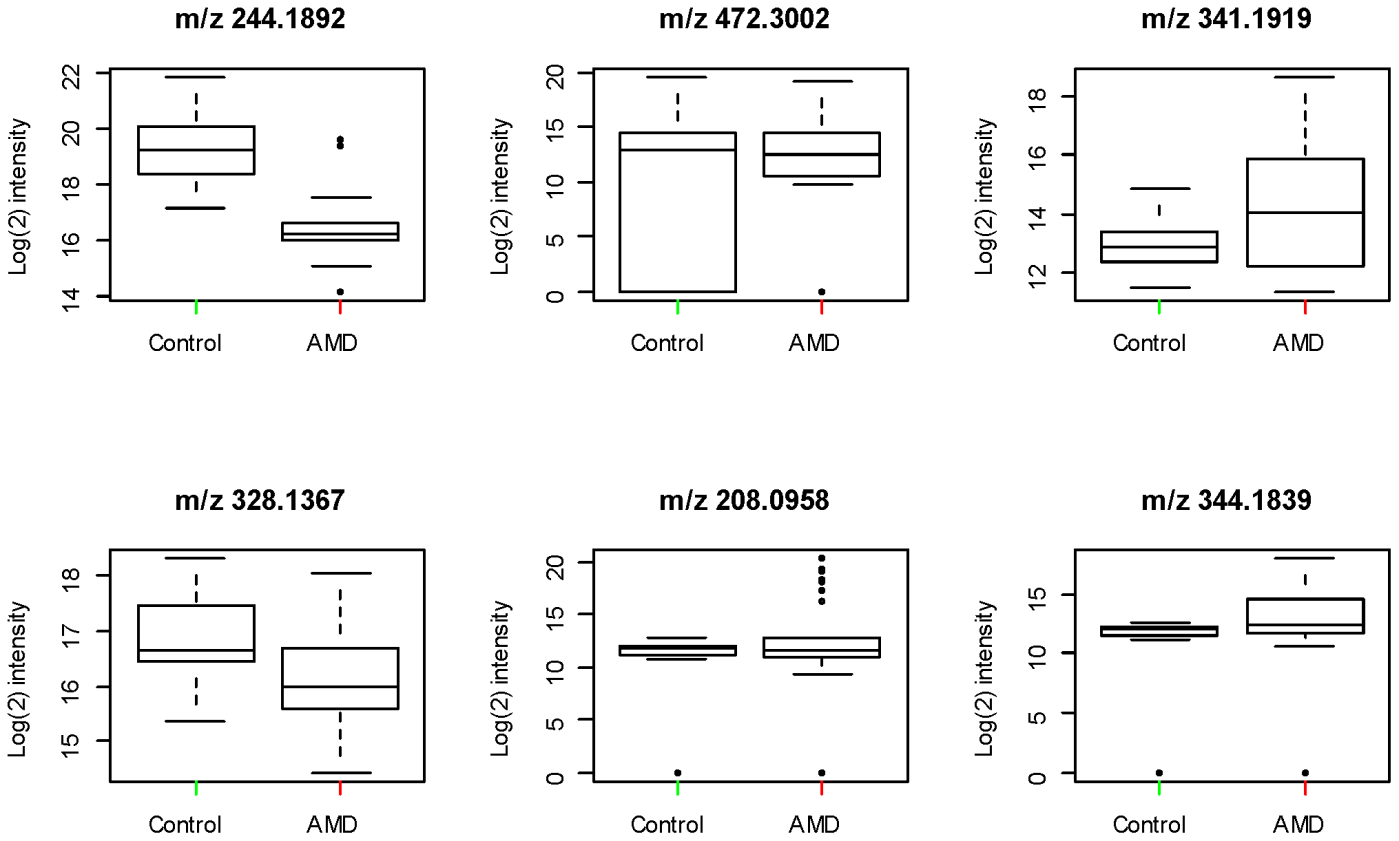
Box-and-whisker plots of selected features comparing the mean and standard error for cases and controls. Plots were examined for each of the 94 features.

Extraction of mass spectral data for the anion exchange chromatography with apLCMS yielded 1680 *m/z* features defined by high-resolution *m/z*, retention time, and ion intensity. Features present in less than 50% of analyses were excluded, leaving 1168 features. In a metabolome-wide association study (MWAS) of the 45 subjects, 94 unique metabolic features significantly differed between the two groups using FDR (q = 0.05) (**Table S1A**). **Figure 1** depicts a Manhattan plot of –logp for each metabolite expressed as a function of the *m/z*, with the 94 features appearing above the broken line indicating significance level. Box-and-whisker plots comparing the mean and standard error for metabolite levels of NVAMD patients and controls were examined for each of the 94 features. Examples of six of these plots are shown in **Figure 2**. The plots for different features suggested that log transformations might improve analyses of individual metabolites. FDR of log2 transformed data resulted in 132 features at q = 0.2, 74 features at q = 0.1, and 39 features at q = 0.05 (**Table S1B–D**). Of the 132 features obtained with log2-transformed data at q = 0.2, 40 intersected the list of 94 features obtained without transformation (**Table S2**). The discriminatory characteristics of the set of the features obtained using the non-transformed data and transformed data were evaluated using 10-fold and leave-one-out cross-validation approaches and a Support Vector Machine (SVM) classifier [23]. Features selected using the non-transformed data at q = 0.05 gave similar classification accuracies to analysis with log2 transformation (**Table S3**). These results show no clear advantage to transformation prior to analysis, but the 40 features intersecting the non-transformed and transformed data were analyzed in order to ensure significance. Many features were found to discriminate NVAMD patients from similarly-aged controls both before and after statistical correction, but the set of 94 features, along with the group of 40 intersecting features, became the focus of further analysis.

### Associations among the Metabolic Features

Correlation analysis is helpful in identifying metabolites that are linked by common metabolic enzymes or transporters. Correlation may occur as a result of biological variability, systematic differences in subject selection or sample collection, or generation of multiple ions from the same chemical during electrospray ionization-mass spectrometry. Correlation analysis of the 94 discriminatory features showed that many of the significant features were associated with each other, but few were strongly associated (**Figure S1**). For more detailed examination, we selected the 40 features that significantly differed between NVAMD and controls both without transformation and following log2 transformation (**Table S2**). Based upon empirical evidence that *m/z* features derived from the same unique chemical entity often correlate with r>0.81 (Y. Park, K. Lee, D.P. Jones, unpublished observation), we also examined any of the total 1168 features having r>0.81 for correlation with these 40 features. A group of 21 features was shown to have one or more correlation; further association within the group was demonstrated by seventeen of these features, which formed four clusters (**Table 1**). Thus, the features that discriminate NVAMD from controls include at least three types of metabolites: ones that do not strongly associate with other features, ones that are likely to be different forms of the same chemical, and ones that are likely to have a biological basis for association.

**Table 1:** Clusters of discriminating *m/z* features (n = 21*) and their respective correlated features at r>0.81.

Cluster	*m/z*	Correlated *m/z* Features
1	186.221	308.857, 376.844, 324.831, 392.818, 347.139, 289.945, 213.909, 243.037, 353.103, 344.184, 348.155, 341.192, 214.062
1	208.096	289.945, 353.103, 341.192, 328.192, 229.096
1	310.187	323.093, 243.037
1	328.192	289.945, 322.189
1	341.192	289.945, 229.096, 208.096, 310.187
1	341.192	213.909, 186.221
1	344.184	229.096, 186.221, 213.909
1	353.103	324.831, 376.844, 308.857, 213.909
2	448.303	449.306, 450.318
2	449.306	448.303, 450.318
2	450.318	451.322, 452.326, 472.300, 414.297, 433.311, 432.308, 473.303, 415.301, 448.303, 453.328, 449.306
2	472.300	451.322, 450.318, 452.326, 414.297, 473.303, 433.311, 415.301, 432.308, 453.328, 434.315
2	656.792	646.762, 147.977, 642.815, 590.801, 588.804, 640.818, 648.760, 658.788, 567.106, 708.806, 672.767, 557.148, 698.777, 551.166, 579.145, 306.048, 630.790, 632.786, 578.776
3	244.189	245.193, 453.366
3	245.193	244.189, 453.366
4	328.137	334.157
4	334.157	328.137
Independent	220.556	223.566, 171.552, 306.048, 551.166, 312.068, 174.562
Independent	293.586	293.084, 290.074, 386.626, 383.616, 386.126, 385.624, 383.114, 200.043, 203.053
Independent	322.189	232.051
Independent	416.211	476.262, 437.190, 432.236, 415.209, 237.116

* Selected from the 40 *m/z* features produced by the intersection of FDR analyses of non-transformed (q = 0.05) and log2 transformed data (q = 0.2).

### Orthogonal Partial Least Squares-discriminant Analysis

**Figure 3:**
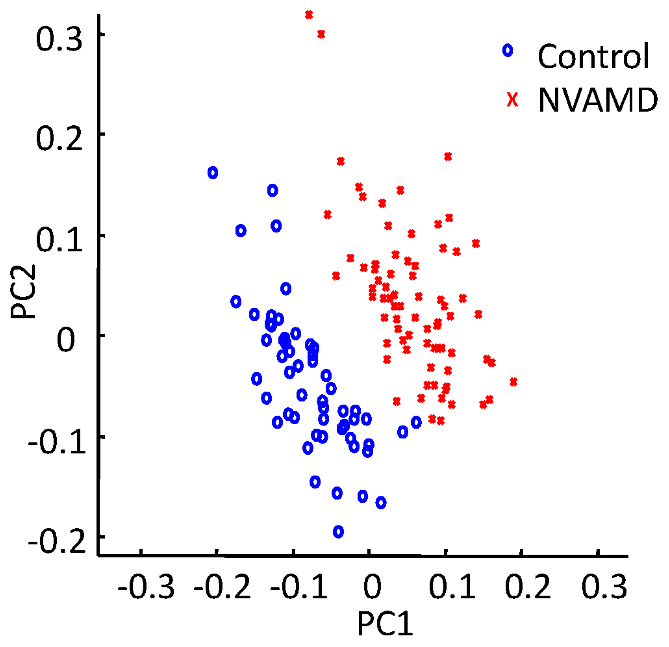
Separation of NVAMD from controls using OPLS-DA.

**Figure 4:**
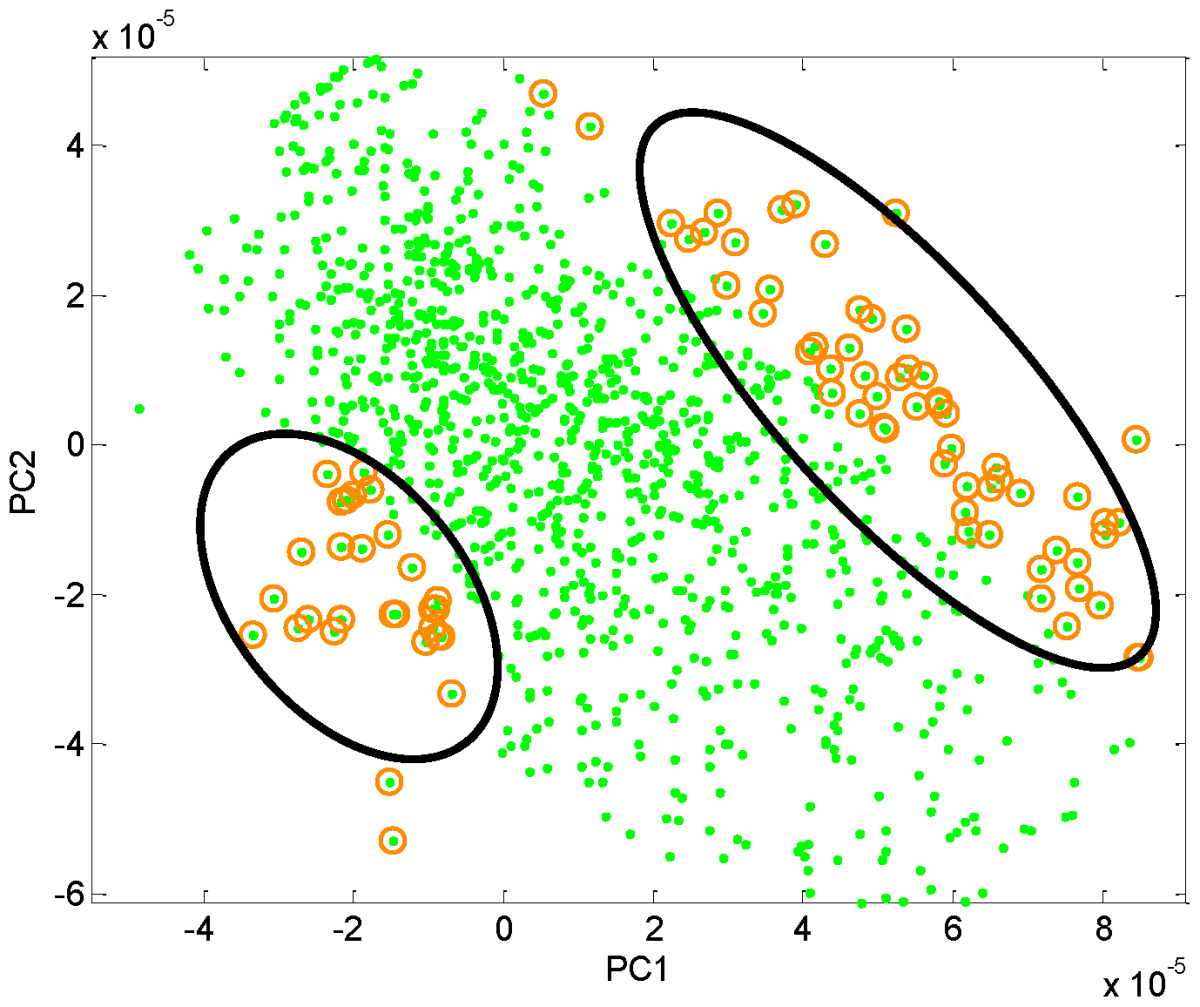
The top 5% of features accounting for 95% separation of NVAMD and controls by OPLS-DA. Green dots represent features that show 95% separation between cases and controls; the gold circles denote the top 5% that contribute to differentiating NVAMD patients (right oval) from controls (left oval).

**Figure 5:**
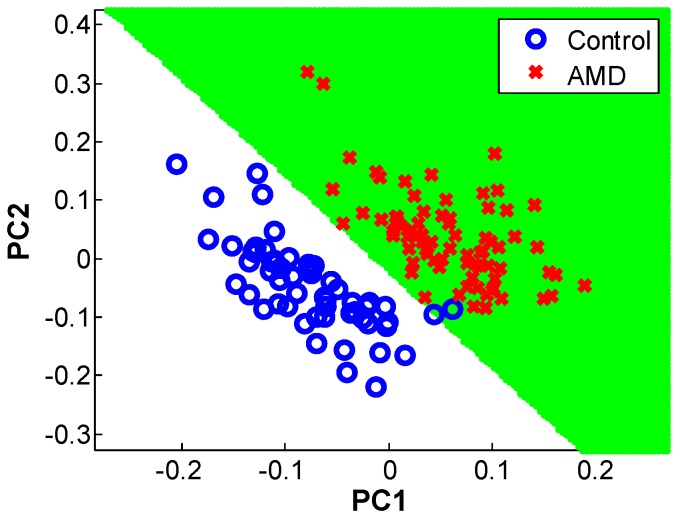
52 features gave >99.1% correct classification by linear discriminant analysis.

We used OPLS-DA to identify features that contribute in a group-wise manner to separation of NVAMD and controls. After removal of the 1^st^ orthogonal component (19.6% of variation), the 1^st^ and 2^nd^ predictive components (38.9% and 34.1% of variation, respectively) largely separated NVAMD from controls (**Figure 3**). We used Principal Component Loading Statistics (PCLS) to identify the top 5% of features that account for 95% separation of NVAMD and controls by OPLS-DA (**Figure 4**). Of these features, 52 gave >99.1% correct classification by linear discriminant analysis (**Figure 5**). These 52 features included 43% of the 94 original non-transformed discriminatory features and 50% of the 40 features intersecting the analysis of the non-transformed and log2 transformed data. Ten features of the 52 were associated with one of the four clusters of features described in **Table 1**. Together, the results show that the metabolic profiles of NVAMD patients and controls differed in contents of features that varied independently and also in content of features that varied with group-wise character.

### Mapping Features to Metabolites and Metabolic Pathways using KEGG Database

High-resolution metabolomics as applied in the current study includes ions formed by electrospray ionization in the positive mode and, therefore, is a selective sampling of the pan-metabolome. The pan-metabolome includes metabolites of the nutritional metabolome derived from products of the human genome acting upon dietary nutrients, food-derived chemicals, products of the microbiome, products of dietary supplements and pharmaceuticals, products derived from commercial products, along with environmental agents and their metabolites [24]. Previous studies show that over half of the ions detected do not match known chemicals in metabolomics databases, and the large number of features detected in the current analyses precludes systematic structural identification. Consequently, we used database searches to help identify possible chemical and metabolic associations with NVAMD. Based upon operational criteria previously discussed [14], we searched for matches within 9 ppm for H^+^, Na^+^, K^+^, H^+^(-H_2_O), H^+^(2H_2_O); a search of the 94 features that distinguished NVAMD from controls by FDR showed that matches were present for 86 metabolites in numerous KEGG human metabolic pathways (**Figure S2**). Identities of amino acids (e.g., phenylalanine, tyrosine, glutamine, aspartate) were confirmed using criteria of coelution with authentic standards and ion dissociation (MS/MS) spectra [7], [14], [20], [21]. Other matches for sulfur amino acid metabolites, several nucleotides, sugars, lipids, and steroid metabolites were present but tend to have multiple isomers that are not distinguished and/or do not have available standards. Matches to several vitamins and coenzymes were also present. From these data, we conclude that the plasma metabolic features that differ between NVAMD and controls include a diverse set of identified and non-identified metabolites.

### Analysis of 40 Features Discriminating NVAMD and Controls using the Metlin Metabolomics Database

The correlations within the group of 40 intersecting features formed four clusters (**Table 1**). Each cluster, along with the features with which each cluster correlated, were searched for matches in the Metlin database corresponding to M+H^+^, M+Na^+^, M+H^+^(-H_2_O), M+H^+^(2H_2_O) forms. The results (**Table 2**) exposed a diverse group of metabolites that distinguished NVAMD from controls by presenting as either increased or decreased in the NVAMD population. The metabolites increased in NVAMD were all features of Cluster 1 and included matches to di- and tripeptides, modified amino acids, and natural products. Correlated features matched to modified amino acids, di- and tripeptides, pentachlorocyclohexanol, and pentachlorodibenzodioxin. Because Cluster 1 included features that were different between NVAMD and controls by statistical criteria and were also among the top 5% of metabolites accounting for 95% separation by OPLS-DA, the data suggest that the metabolic phenotype of NVAMD is a complex network of metabolites including peptides, modified amino acids, natural products, and environmental agents.

**Table 2:** Selected *m/z* features (n = 17) discriminating NVAMD and control patients with their respective matches from the Metlin database.

Cluster	*m/z*	Presence in AMD Cohort	Metlin Match	Metlin Matches of Correlated Features
**1**	186.221	Higher	No Match	Pentachlorochyclohexanol; pentachlorodibenzodioxin; tripeptides; modified amino acids
**1**	208.096	Higher	Acetylphenylalanine	Acetyltryptophan; features from Cluster
**1**	310.187	Higher	Dipeptide; Tripeptides	Modified cysteine and alanine acids
**1**	328.192	Higher	Sethoxydim (herbicide)	Tripeptides
**1**	341.192	Higher	Tripeptides	Acetyltryptophan; features from Cluster
**1**	341.192	Higher	Tripeptides	No Matches
**1**	344.184	Higher	Tripeptides	Acetyltryptophan
**1**	353.103	Higher	Flavones; halofenozide	Pentachlorochyclohexanol, pentachlorodibenzodioxin
**2**	448.303	Lower	Glycocholic acid	Features from Cluster 2
**2**	449.306	Lower	Vitamin D-related metabolites; phytochemicals	Features from Cluster 2
**2**	450.318	Lower	Glycodeoxycholic acid+H+; Glycoursodeoxycholic acid+H+	Vitamin D-related metabolites; ions derived from sphingofungin A; terpenoid; features from Cluster
**2**	472.300	Lower	Glycodeoxycholic acid+Na+; Glycoursodeoxycholic acid+Na+	Vitamin D-related metabolites; ions derived from sphingofungin A; terpenoid; features from Cluster
**2**	656.792	Lower	No Match	Phytochemicals; glutamate metabolites
**3**	244.189	Lower	No Match	Feature from Cluster; one unmatched feature
**3**	245.193	Lower	Senecrassidiol	Feature from Cluster; one unmatched feature
**4**	328.137	Lower	Didemethylsimmondsin	Feature from Cluster
**4**	334.157	Lower	Dipeptides	Feature from Cluster

The second largest cluster of features, Cluster 2, included features that were significantly different between NVAMD and controls, but none were among the top 5% of features discriminating the groups by OPLS-DA. Therefore, these features may be important for some individuals but not for the overall separation of the groups. These features matched primarily to bile acids and were significantly lower in NVAMD. They correlated with an additional 28 features with r>0.81, which included matches for intermediary metabolites and natural products such as glycodeoxycholic acid and vitamin D-related metabolites. Two features matched ions derived from sphingofungin A, a natural antifungal produced by *Aspergillus* species. Sixteen features did not match any metabolite in the database.

Clusters 3 and 4 contained a smaller number of features and had lower values in NVAMD. In Cluster 3, *m/z* feature 245.193 matched senecrassidiol while *m/z* 244.189 and *m/z* 453.366 did not have matches in Metlin. The features in Cluster 4 matched dipeptides (His, Arg; Trp, Phe) and a metabolite of a natural product, didemethylsimmondsin.

The nineteen features showing no strong correlation with other features were similarly searched for matches (H^+^, Na^+^, H^+^(-H_2_O), H^+^(2H_2_O)) to metabolites in Metlin; eight were found to have no matches. The other eleven were matched to tripeptides, metabolic intermediates, food products, dietary supplement metabolites, pharmaceutical metabolites, environmental agents, and thirteen other metabolites, most of which were natural products. Features that were increased in NVAMD included matches to a tripeptide (292.152) and two environmental agents (222.112, 419.313) while those decreased included three metabolic intermediates (144.101, 346.007, 368.070), a tripeptide (421.159), and three phytochemicals (365.086, 371.055, 421.159).

### Interpretation of Metabolic differences between NVAMD and Controls using MetScape

**Figure 6:**
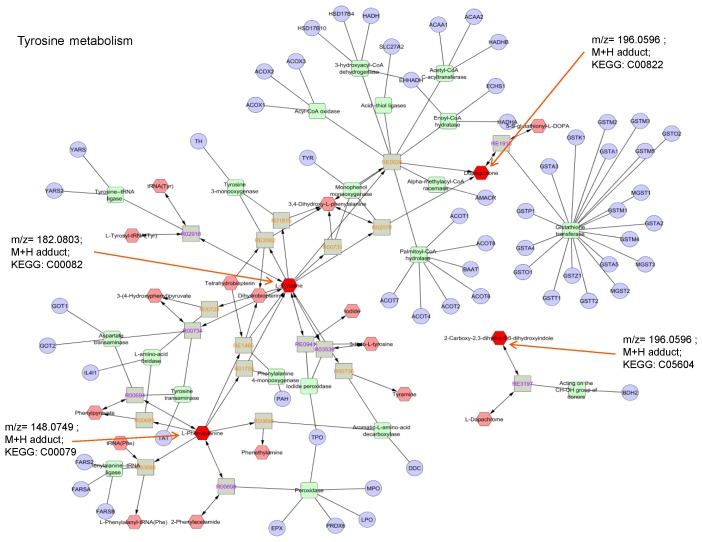
Map of tyrosine metabolism. Metabolites that matched *m/z* features of log2 transformed data at q = 0.2 are designated by orange arrows. When compared to controls, NVAMD patients showed lower levels of *m/z* 148.0749 but higher levels of *m/z* 182.0803 and *m/z* 196.0596. Note that the methods used do not discriminate between the two metabolites matching the *m/z* 196.0596 M+H adduct.

MetScape is a plug-in for Cytoscape, an open source software platform for visualizing complex networks. MetScape analysis of the 94 features identified multiple associated pathways, including fructose and mannose metabolism, galactose metabolism, pentose phosphate pathway and tyrosine metabolism (**Table S4**). In addition, Metscape identified bile acid biosynthesis, glycosphingolipids, thiamine metabolism, and two pathways previously associated with AMD: the urea cycle, including metabolism of arginine, proline, glutamate, aspartate and asparagine, as well as CoA biosynthesis from pantothenate [25]. Analysis of the compound-reaction-enzyme-gene network analysis for 132 discriminatory features using log2 transformed data and FDR at q = 0.2 confirmed the network associations for the tyrosine (**Figure 6**) and urea metabolism (**Figure S3**) pathways, as well as nicotinamide, pyrimidine, lysine and methionine plus cysteine (not shown). These results indicate that the metabolites discriminating NVAMD and controls map to carbohydrate, amino acid, and coenzyme metabolites required for nitrogen balance and energy metabolism.

## Discussion

This metabolome-wide association study (MWAS) demonstrates the ability of LC-FTMS to pinpoint both individual metabolites and metabolic pathways that are likely to play an important role in AMD pathophysiology. Our evaluation of 26 NVAMD patients and 19 controls closely matched in age and general health identified 94 unique metabolites that significantly differed between the groups using FDR (q = 0.05). The subset of 40 metabolites that intersected both non-transformed and log2 transformed data were selected for detailed investigation. Correlation analysis of these 40 features showed that over half associated with other discriminating metabolites. OPLS-DA was performed to uncover features that contribute in a group-wise manner to the separation of cases and controls. This analysis revealed a panel of 52 metabolites that showed >99.1% separation of NVAMD from controls. These data suggest that comprehensive metabolomics can expose both individual metabolites and metabolic profiles that discern disease status.

We used the Metlin and KEGG databases to identify specific metabolites that distinguished NVAMD from controls. Discriminating *m/z* features that highly correlated with other features fell into four clusters containing a total of seventeen metabolites. These seventeen features yielded consistent Metlin matches to di- and tripeptides, covalently modified amino acids, bile acids, and vitamin D-related metabolites. Since these clusters involve metabolites that are likely to have a biological basis for association, their matches may reveal more consistent and functional information regarding AMD disease status than single metabolite markers. For example, the features in Cluster 1 matching to small peptides and modified amino acids were among the top 5% of metabolites accounting for 95% separation by OPLS-DA, denoting their importance.

The *m/z* features matching tripeptides and dipeptides in Cluster 1 had higher levels in NVAMD patients than controls. Increased levels of small peptides in the plasma could be the result of excessive proteosomal activity, abnormalities in removal of peptides by peptidases or altered function of peptide transporters. For example, the peptide transporter PEPT2 is expressed in peripheral tissues and the central nervous system, including the Mϋller cells of the retina [26]. Studies of *Pept2* knockout mice demonstrated that aberrant Pept2 function can lead to significant alterations in dipeptide disposition [27].

Additional *m/z* features in Cluster 1 matched to modified amino acids such as acetylphenylalanine and acetyltryptophan. Acetylphenylalanine is a hazardous metabolite of phenylalanine and is elevated in phenylketonuria due to lack of phenylalanine hydroxylase, the enzyme that converts phenylalanine to tyrosine [28]. In a previous metabolomics study, acetylphenylalanine levels were found to be elevated in patients with renal cell carcinoma [28]. Acetylphenylalanine is typically bound to albumin in the plasma and excreted into the urine via the organic ion transporter [29]. Low serum albumin levels, which have been previously associated with neovascular AMD [30], or abnormal renal ion transporter function could account for the increased levels of the modified amino acids in the NVAMD patients of this study. Metabolomic analysis of urine in NVAMD and control patients would help determine if the higher levels of modified amino acids are due to abnormal production or reduced excretion.

The *m/z* features from Cluster 2, along with their correlated features, matched multiple bile acids and were found to be decreased in NVAMD patients compared to controls. Specifically, one *m/z* feature in the cluster matched glycocholic acid while two features matched glycodeoxycholic acid and glycoursodeoxycholic acid. Through activation of multiple signaling pathways, bile acids are critical to the regulation of triglyceride, cholesterol, glucose, and energy homeostasis [31]. Low bile acid levels that lead to disruption of any of these metabolic pathways may affect AMD pathophysiolology. Additionally, glycoursodeoxycholic acid (GUDCA) has been shown to act as an antioxidant by protecting neurons against unconjugated bilirubin-induced oxidative stress [32]. Interestingly, tauroursodeoxycholic acid (TUDCA) has been shown to protect photoreceptors from cell death in murine models of retinitis pigmentosa [33] and retinal detachment [34], as well as to suppress choroidal neovascularization in a laser-treated rat model [35]. Thus, bile acids might serve global anti-apoptotic or anti-angiogenic roles that are critical to retinal function.

A single *m/z* feature from Cluster 2 with lower levels in NVAMD patients compared to controls, along with multiple correlated features, matched to vitamin D-related metabolites. Vitamin D is thought to affect immune modulation and perhaps even slow or prevent diseases with inflammatory etiologies [36]. Lower serum vitamin D levels were associated with AMD in an evaluation of patients in the third National Health and Nutrition Examination Survey (NHANES) [37], as well as in post-menopausal women in the Carotenoids in Age-Related Eye Disease Study (CAREDS) [38]. In other studies, vitamin D levels were shown to be lower in patients with late AMD [39] and were associated with poorer visual acuity in older adults [40]. The lower levels of vitamin D-related metabolites seen in this study are consistent with these findings and suggest that vitamin D may be protective against AMD. Previous laboratory studies support a role in the prevention of neovascularization, providing evidence that 1α,25-dihydroxyvitamin D inhibits abnormal angiogenesis *in vitro* and *in vivo* [41].

Three *m/z* features from the four clusters and several of the correlated features had no match in the Metlin database using the predefined search criteria (Table 2). This suggests that some environmental metabolites measured by high-resolution metabolomics analysis have not yet been identified, showing that this technique could prove advantageous over strictly targeted methods in a comprehensive analysis of metabolic profiles.

In our pathway analyses, the Metscape database provided results that complemented findings using the KEGG database. KEGG matches for the 94 discriminatory features included amino acids linked to tyrosine metabolism and the urea cycle, such as phenylalanine, tyrosine, glutamine, and aspartate; Metscape pathway analysis identified seventeen affected metabolic pathways, including tyrosine and urea metabolism. Individual metabolites in the tyrosine pathway that differed between NVAMD and controls included tyrosine, phenylalanine, and dopaquinone. These pathway results are consistent with the previously found match to the Metlin database identifying the modified amino acid acetylphenylalanine, an abnormal variation of a metabolite in the tyrosine synthesis pathway.

This proof-of-principle study was limited by its sample size. The relatively small number of patients and controls could have allowed a random enrichment of metabolic characteristics in either the NVAMD or control groups and will require extension to larger populations to determine whether these findings represent common metabolic characteristics of NVAMD patients. Multiple steps were taken to increase the probability that the metabolites found to discriminate between NVAMD and control in this study represent true differences in disease status. First, the two populations did not differ in age, gender, smoking status, multivitamin supplement intake, or key comorbidities including diabetes, coronary artery disease, hypertension, hyperlipidemia, and history of cancer. This similarity greatly improves the likelihood that discriminating metabolites are due to AMD and not the result of confounding factors. Second, we focused our correlation analysis on metabolites that distinguished NVAMD from control using both non-transformed and log transformed data, decreasing the chance of data outliers affecting significance. Finally, the considerable overlap of results from analyses designed to identify individual metabolites (FDR) and metabolites that contribute in a group-wise fashion (OPLS-DA) suggests biologic plausibility for these metabolites and pathways. The putative metabolite identifications will need to be confirmed by MS/MS and coelution with authentic standards, and their ability to differentiate NVAMD patients from controls will need to be validated with an independent replication cohort.

The metabolic changes linked with NVAMD in this study could have preceded development of disease or could be a result of disease or treatment status; causality cannot be inferred from this association study. Furthermore, differential metabolite levels between NVAMD patients and controls could be related to metabolic changes at any stage of AMD disease progression. To separate out metabolites specifically related to early AMD or neovascularization, metabolic analyses of these two populations will be required. Studies in which archived plasma samples can be assayed along with longitudinal clinical data may provide information on the chronology and predictive capacity of metabolic changes. Xu *et al.* recently identified modest levels of anti-VEGF agent ranibizumab in serum after intravitreal injection [42]. While the present metabolomics methods are not suitable for detection of ranibizumab, prospective studies could allow investigation of the effects of anti-VEGF treatment as well as AREDS-type oral supplements on plasma metabolites. Additionally, identifying metabolites that are altered in association with known genetic risk factors could allow a better understanding of how genetic associations play a role in disease manifestation. Nevertheless, the results of this study provide a rich set of data with which to move forward in mapping out critical AMD-related metabolites.

The cross-validation analysis suggests that significant metabolic heterogeneity exists within the NVAMD patients. Thus, NVAMD patients who are clinically indistinguishable may actually have very different metabolic characteristics and could potentially be subclassified by metabolic phenotype. Such metabolic subclassification could lead to improved prevention and treatment strategies.

In summary, these data reveal that NVAMD pathophysiology may involve a map of diverse metabolic components that range from peptides, bile acids, and vitamin D to broader pathways like that of tyrosine metabolism. Our results suggest two advantages of a high-resolution metabolomic approach: it can provide relative quantification of a large number of metabolites to facilitate comprehensive analysis of environmental impact on disease status, and, secondly, it can discern metabolic profiles and pathways that distinguish NVAMD cases from controls. Such plasma metabolic phenotyping could improve current diagnostic methods for AMD by substantiating evidence of disease or disease risk prior to clinical manifestation.

## Supporting Information

Figure S1 **Metabolite-Metabolite pairwise correlation heatmap of the 94 discriminatory metabolites between NVAMD and control groups at q = 0.05.** The colors represent the Pearson correlation coefficient; dark red indicating highly positive correlation and dark blue corresponding to highly negative correlation.
(TIF)

Figure S2 **KEGG metabolic pathway analysis with 94 matched features.** The 94 *m/z* features that differed significantly between NVAMD patients and controls using FDR at q = 0.05 were compared to the KEGG Metabolic Pathway Database, revealing 86 individual metabolites (black dots) in multiple pathways that discriminate between NVAMD patients and controls. Note that these are matches to the metabolites based upon accurate mass *m/z* and do not represent confirmed identifications. Approximately 90% of the metabolites in the KEGG human metabolic pathways have unique elemental compositions, and our previous studies [7], [14], [20], [21] with MS/MS and coelution of standards show that 60–80% of matches are correct. However, certain ambiguities exist; for example, UDP-glucose and UDP-galactose are identified as matches, but having identical elemental compositions prevents them from being separated by these methods.
(TIF)

Figure S3 **Maps of urea cycle and relevant amino acid metabolisms.** Metabolites that matched *m/z* features are designated with arrows.
(TIF)

Table S1 (DOCX)

Table S2 (DOCX)

Table S3 (DOCX)

Table S4 (DOCX)
