# Supplementary figures and images for: Metabolome-Wide Association Study of Neovascular Age-Related Macular Degeneration
Source: PLoS One. 2013 Aug 27;8(8):e72737. doi: 10.1371/journal.pone.0072737 (PMC3754980; doi:10.1371/journal.pone.0072737)

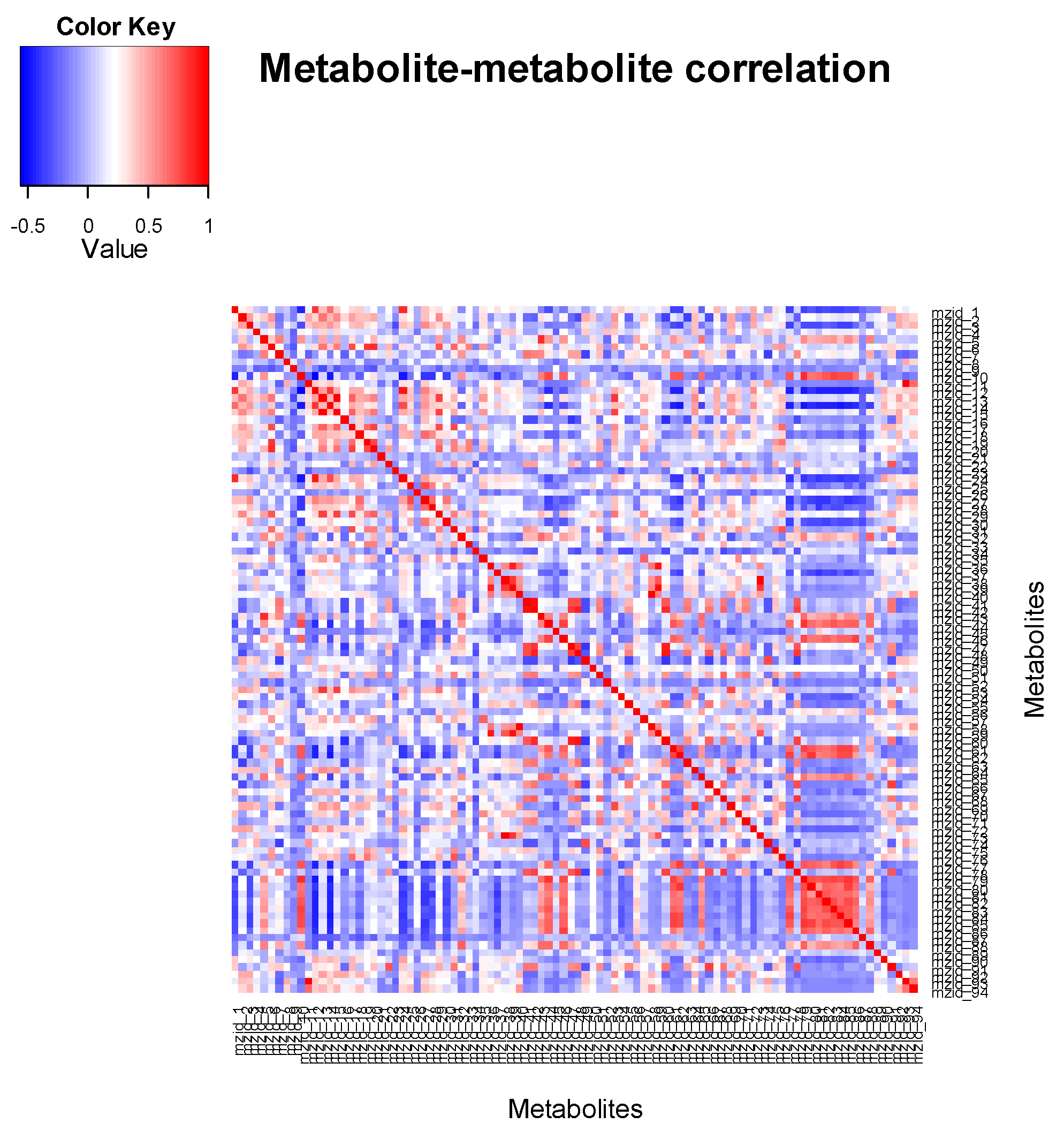

Supplement: Figure S1 — Metabolite-Metabolite pairwise correlation heatmap of the 94 discriminatory metabolites between NVAMD and control groups at q = 0.05. The colors represent the Pearson correlation coefficient; dark red indicating highly positive correlation and dark blue corresponding to highly negative correlation. (TIF) [file pone.0072737.s001.tif]

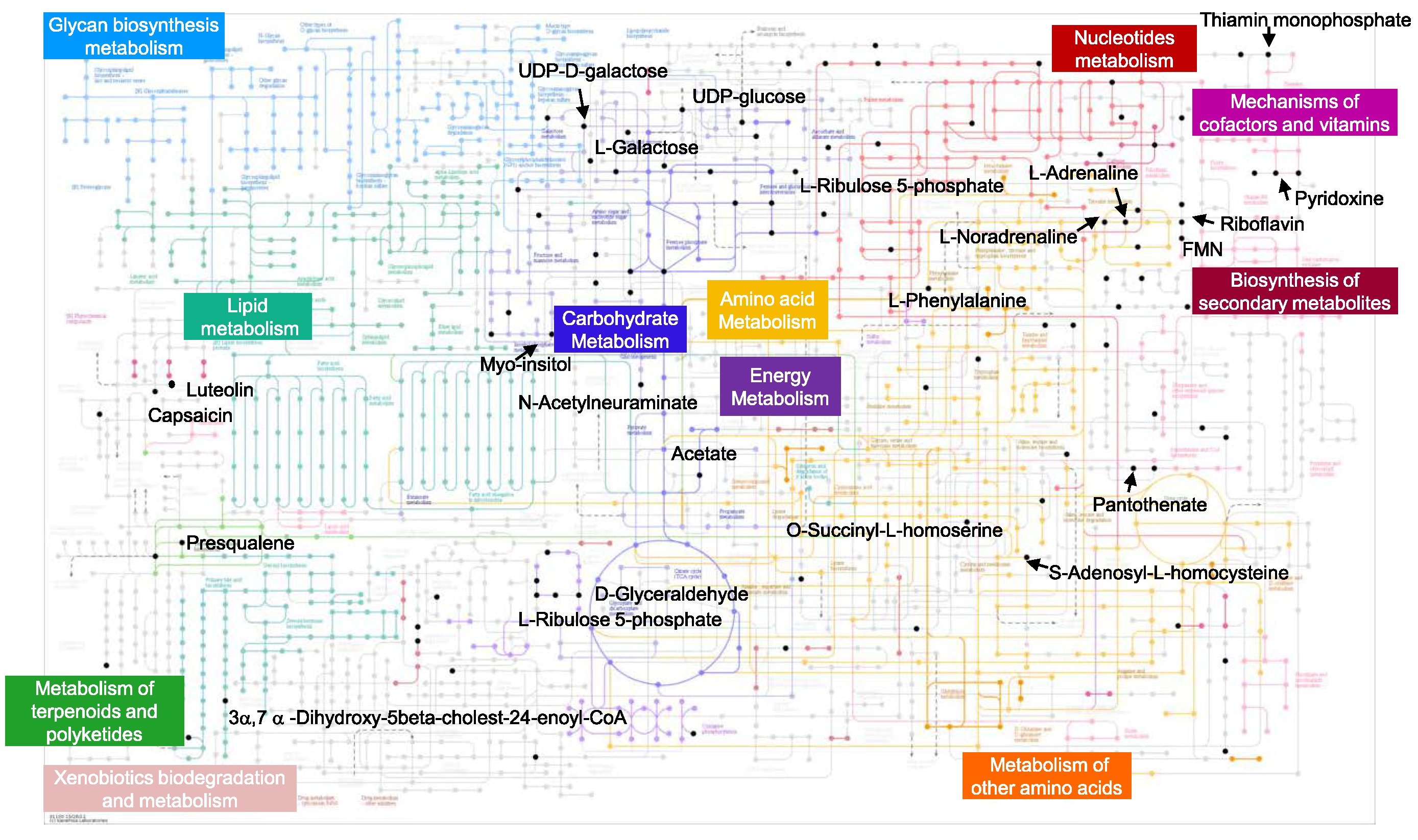

Supplement: Figure S2 — KEGG metabolic pathway analysis with 94 matched features. The 94 m/z features that differed significantly between NVAMD patients and controls using FDR at q = 0.05 were compared to the KEGG Metabolic Pathway Database, revealing 86 individual metabolites (black dots) in multiple pathways that discriminate between NVAMD patients and controls. Note that these are matches to the metabolites based upon accurate mass m/z and do not represent confirmed identifications. Approximately 90% of the metabolites in the KEGG human metabolic pathways have unique elemental compositions, and our previous studies [7], [14], [20], [21] with MS/MS and coelution of standards show that 60–80% of matches are correct. However, certain ambiguities exist; for example, UDP-glucose and UDP-galactose are identified as matches, but having identical elemental compositions prevents them from being separated by these methods. (TIF) [file pone.0072737.s002.tif]

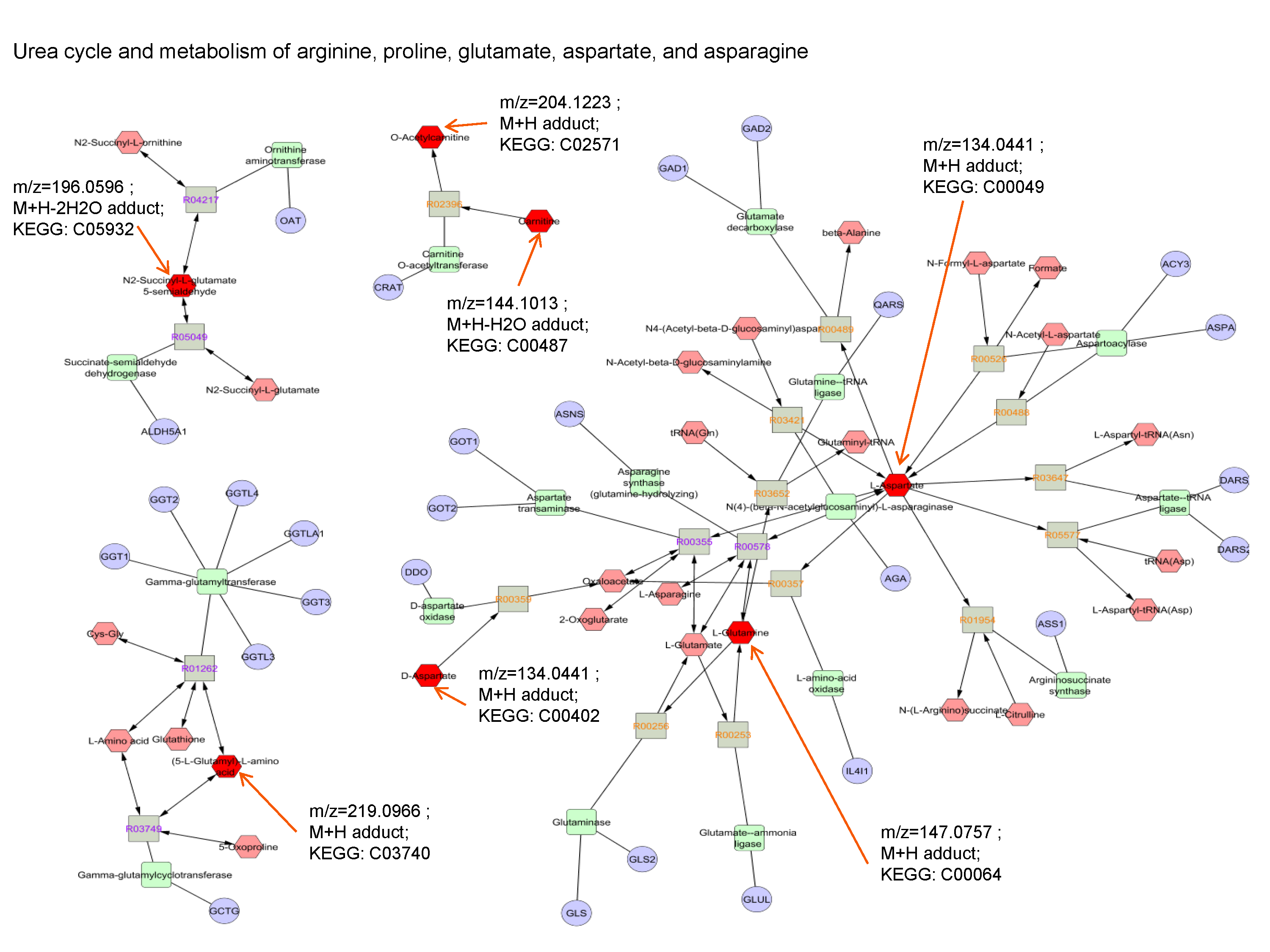

Supplement: Figure S3 — Maps of urea cycle and relevant amino acid metabolisms. Metabolites that matched m/z features are designated with arrows. (TIF) [file pone.0072737.s003.tif]
