# Supplementary material for: Metabolome-Wide Association Study of Neovascular Age-Related Macular Degeneration
Source: PLoS One. 2013 Aug 27;8(8):e72737. doi: 10.1371/journal.pone.0072737 (PMC3754980; doi:10.1371/journal.pone.0072737)
Supplement: Table S1 — (DOCX) [file pone.0072737.s004.docx]

**Table S1.** Features detected as significant by False Discovery Rate between 26 neovascular age-related macular degeneration patients and 19 similarly-aged controls.

**A. Using intensity values without transformation of data, q= 0.05**

| ***m/z*** | **time (s)** | **Control Mean** | **Control SD** | **AMD Mean** | **AMD SD** |
| --- | --- | --- | --- | --- | --- |
| 98.984 | 550 | 49191 | 34379 | 23813 | 30244 |
| 144.101 | 103 | 331402 | 449011 | 128772 | 172579 |
| 151.035 | 80 | 270582 | 172023 | 187999 | 113894 |
| 157.083 | 114 | 323191 | 140249 | 235983 | 134922 |
| 166.085 | 103 | 940510 | 258732 | 799848 | 201419 |
| 186.221 | 329 | 2077 | 2717 | 91286 | 180950 |
| 197.040 | 80 | 213411 | 127821 | 307968 | 235405 |
| 203.053 | 108 | 254884 | 99027 | 207925 | 87758 |
| 208.039 | 84 | 561685 | 561537 | 902467 | 690245 |
| 208.096 | 340 | 2552 | 3111 | 119009 | 287348 |
| 212.042 | 504 | 64604 | 52053 | 36006 | 41728 |
| 213.017 | 87 | 734144 | 385027 | 1157905 | 919424 |
| 216.081 | 146 | 2976 | 4508 | 41339 | 82907 |
| 218.995 | 86 | 5379 | 7727 | 16256 | 24963 |
| 219.085 | 386 | 128776 | 140593 | 65404 | 96988 |
| 219.097 | 141 | 118832 | 84041 | 86852 | 59891 |
| 220.556 | 108 | 43919 | 58887 | 19256 | 31652 |
| 222.112 | 449 | 53662 | 99006 | 147025 | 207275 |
| 243.037 | 106 | 88713 | 38269 | 189508 | 169598 |
| 244.189 | 147 | 832445 | 836177 | 78238 | 40736 |
| 245.193 | 145 | 90228 | 110175 | 1670 | 3129 |
| 261.130 | 105 | 134889 | 123272 | 71313 | 90754 |
| 261.960 | 87 | 60932 | 80447 | 33436 | 52577 |
| 266.171 | 144 | 50591 | 77833 | 1043 | 2545 |
| 275.933 | 552 | 175970 | 78576 | 80507 | 82323 |
| 276.118 | 161 | 3441695 | 1606092 | 2575912 | 1318365 |
| 276.985 | 526 | 130268 | 60580 | 63203 | 60752 |
| 277.121 | 161 | 350457 | 177460 | 258747 | 147402 |
| 278.039 | 113 | 112331 | 77050 | 67721 | 58118 |
| 284.126 | 169 | 138673 | 118621 | 137795 | 115900 |
| 284.126 | 466 | 300328 | 307040 | 177336 | 129183 |
| 287.067 | 113 | 314257 | 120927 | 241765 | 110079 |
| 292.152 | 400 | 2302 | 2589 | 29568 | 61544 |
| 293.096 | 474 | 1179433 | 775014 | 749380 | 517181 |
| 293.586 | 108 | 140472 | 73948 | 104440 | 58968 |
| 296.095 | 110 | 1240712 | 389107 | 1022302 | 402965 |
| 302.213 | 119 | 59075 | 53181 | 36208 | 27153 |
| 305.085 | 382 | 39662 | 40038 | 86484 | 121175 |
| 305.155 | 104 | 137623 | 116609 | 75103 | 57243 |
| 310.187 | 141 | 3413 | 3116 | 28385 | 54808 |
| 320.869 | 94 | 134095 | 51487 | 110354 | 51865 |
| 322.189 | 460 | 8494 | 22523 | 89294 | 175649 |
| 325.010 | 118 | 150983 | 73603 | 115695 | 59377 |
| 325.209 | 473 | 98121 | 88881 | 200846 | 186266 |
| 328.137 | 131 | 125171 | 79373 | 84835 | 66928 |
| 328.192 | 156 | 2531 | 3146 | 31084 | 61084 |
| 332.882 | 550 | 93636 | 53109 | 35893 | 46478 |
| 333.143 | 169 | 70235 | 72353 | 16791 | 32149 |
| 334.157 | 132 | 102775 | 53878 | 68473 | 44217 |
| 337.129 | 115 | 88945 | 52319 | 50128 | 44526 |
| 341.192 | 187 | 2923 | 2492 | 61804 | 121676 |
| 341.192 | 472 | 8584 | 12350 | 71130 | 127437 |
| 344.012 | 526 | 274231 | 113614 | 201348 | 128731 |
| 344.184 | 143 | 3424 | 3078 | 38334 | 76429 |
| 345.029 | 358 | 193650 | 95069 | 289855 | 195090 |
| 346.007 | 529 | 5407663 | 2240680 | 3812961 | 2029622 |
| 347.011 | 478 | 442116 | 201224 | 325570 | 187175 |
| 348.155 | 124 | 48323 | 50022 | 100713 | 124436 |
| 353.103 | 152 | 3442 | 4395 | 34975 | 74192 |
| 356.068 | 133 | 15908 | 17054 | 33402 | 34952 |
| 362.171 | 121 | 157868 | 86187 | 257343 | 245214 |
| 365.086 | 98 | 174449 | 103399 | 127171 | 61088 |
| 366.071 | 123 | 123751 | 88055 | 82555 | 57011 |
| 368.070 | 113 | 60166 | 41803 | 37097 | 33662 |
| 370.910 | 126 | 17621 | 27464 | 7399 | 11312 |
| 371.055 | 154 | 27566 | 49125 | 5422 | 17424 |
| 371.077 | 168 | 629171 | 262009 | 470244 | 298008 |
| 381.047 | 111 | 48452 | 46085 | 28447 | 29261 |
| 381.984 | 528 | 1099845 | 409204 | 837641 | 531873 |
| 387.043 | 115 | 197582 | 91131 | 145693 | 74273 |
| 391.104 | 106 | 21942 | 31358 | 41636 | 57122 |
| 395.154 | 106 | 195795 | 61776 | 264646 | 132714 |
| 405.098 | 114 | 68507 | 68107 | 33961 | 34486 |
| 413.127 | 171 | 25410 | 31868 | 96566 | 172718 |
| 416.035 | 117 | 105839 | 66382 | 74302 | 54765 |
| 416.211 | 448 | 5761 | 11286 | 28583 | 67815 |
| 419.313 | 488 | 103604 | 95729 | 189074 | 159399 |
| 420.156 | 148 | 200711 | 126393 | 125528 | 119119 |
| 421.159 | 147 | 150513 | 100927 | 80321 | 87155 |
| 432.043 | 534 | 68655 | 66231 | 26079 | 41849 |
| 433.311 | 449 | 311469 | 564252 | 115949 | 122187 |
| 443.982 | 546 | 259571 | 145411 | 128720 | 143720 |
| 448.303 | 423 | 612536 | 979519 | 231263 | 230558 |
| 449.306 | 428 | 133849 | 260285 | 28949 | 43929 |
| 450.318 | 468 | 5799295 | 8661201 | 2650576 | 2336635 |
| 466.313 | 341 | 3050911 | 5300098 | 773900 | 730780 |
| 467.317 | 378 | 775557 | 1417597 | 159002 | 180176 |
| 472.300 | 470 | 95397 | 214327 | 17686 | 38842 |
| 549.051 | 114 | 53883 | 77920 | 34884 | 42109 |
| 551.166 | 104 | 48861 | 64810 | 35638 | 42713 |
| 580.774 | 93 | 77026 | 101149 | 74637 | 77026 |
| 590.801 | 94 | 104793 | 124385 | 97421 | 98114 |
| 624.843 | 92 | 292622 | 389778 | 296189 | 345320 |
| 640.818 | 93 | 243092 | 305029 | 218708 | 286577 |
| 656.792 | 93 | 94771 | 121693 | 65697 | 108927 |

**B. Using intensity values with log2 transformation of data, q= 0.2**

| ***m/z*** | **time (s)** | **Control Mean** | **Control SD** | **AMD Mean** | **AMD SD** |
| --- | --- | --- | --- | --- | --- |
| 101.416 | 121 | 505049 | 435687 | 593406 | 320168 |
| 102.066 | 112 | 50481 | 34125 | 82237 | 80967 |
| 121.071 | 109 | 16928 | 24495 | 35458 | 42141 |
| 134.044 | 473 | 212541 | 224627 | 153796 | 166281 |
| 144.101 | 103 | 331402 | 449011 | 128772 | 172579 |
| 146.049 | 156 | 307620 | 234300 | 183606 | 200307 |
| 147.076 | 104 | 830513 | 139248 | 739138 | 247728 |
| 148.075 | 494 | 98429 | 114271 | 70669 | 100491 |
| 148.093 | 153 | 190744 | 187213 | 165307 | 193881 |
| 150.058 | 104 | 121844 | 60088 | 104763 | 46053 |
| 157.083 | 114 | 323191 | 140249 | 235983 | 134922 |
| 174.562 | 105 | 24892 | 46771 | 12161 | 22907 |
| 182.057 | 101 | 42508 | 58356 | 22189 | 41814 |
| 182.080 | 459 | 230042 | 235241 | 250175 | 239878 |
| 186.221 | 329 | 2077 | 2717 | 91286 | 180950 |
| 190.911 | 96 | 540379 | 203317 | 476734 | 225843 |
| 196.060 | 362 | 32370 | 73119 | 126527 | 312913 |
| 204.122 | 99 | 293531 | 127056 | 244544 | 104453 |
| 208.096 | 340 | 2552 | 3111 | 119009 | 287348 |
| 216.081 | 493 | 1368 | 2267 | 27718 | 69001 |
| 219.085 | 386 | 128776 | 140593 | 65404 | 96988 |
| 219.097 | 400 | 59328 | 104409 | 19815 | 40126 |
| 220.556 | 108 | 43919 | 58887 | 19256 | 31652 |
| 222.056 | 329 | 14650 | 51239 | 167768 | 363194 |
| 222.112 | 449 | 53662 | 99006 | 147025 | 207275 |
| 222.196 | 150 | 240889 | 214208 | 252048 | 361362 |
| 222.196 | 471 | 246905 | 261913 | 281320 | 480777 |
| 223.095 | 338 | 1282407 | 1420242 | 903231 | 1119482 |
| 224.158 | 146 | 45444 | 76616 | 17110 | 52568 |
| 227.982 | 398 | 41628 | 151031 | 105622 | 236495 |
| 229.140 | 477 | 77686 | 84239 | 113757 | 79703 |
| 232.051 | 482 | 16713 | 37919 | 66313 | 143021 |
| 239.090 | 145 | 431108 | 639378 | 190509 | 291712 |
| 241.030 | 111 | 404346 | 116968 | 482572 | 278486 |
| 242.924 | 96 | 7301718 | 1468319 | 6604326 | 1652795 |
| 243.037 | 106 | 88713 | 38269 | 189508 | 169598 |
| 244.189 | 147 | 832445 | 836177 | 78238 | 40736 |
| 245.193 | 145 | 90228 | 110175 | 1670 | 3129 |
| 255.231 | 526 | 7705 | 19010 | 7603 | 13424 |
| 256.109 | 114 | 141882 | 136719 | 164764 | 136841 |
| 257.112 | 194 | 2879 | 4654 | 56883 | 146066 |
| 258.898 | 96 | 1934547 | 598958 | 1674621 | 643953 |
| 261.010 | 115 | 74857 | 80839 | 100081 | 109450 |
| 263.012 | 104 | 103356 | 53139 | 136738 | 88851 |
| 267.030 | 115 | 53406 | 70894 | 82029 | 80709 |
| 270.932 | 68 | 2161 | 3067 | 184600 | 667134 |
| 278.039 | 113 | 112331 | 77050 | 67721 | 58118 |
| 279.094 | 161 | 117228 | 186023 | 130882 | 159173 |
| 283.003 | 118 | 25008 | 47784 | 32563 | 43680 |
| 285.070 | 252 | 38512 | 76227 | 30031 | 64205 |
| 287.063 | 344 | 7780 | 24995 | 3298 | 17754 |
| 288.137 | 129 | 35524 | 31246 | 20516 | 34339 |
| 292.152 | 400 | 2302 | 2589 | 29568 | 61544 |
| 293.586 | 108 | 140472 | 73948 | 104440 | 58968 |
| 294.090 | 120 | 52246 | 51804 | 38727 | 36256 |
| 294.153 | 115 | 85793 | 53404 | 67953 | 71885 |
| 295.112 | 128 | 35554 | 25603 | 27979 | 43178 |
| 296.596 | 108 | 40678 | 25542 | 31945 | 26852 |
| 310.187 | 141 | 3413 | 3116 | 28385 | 54808 |
| 310.187 | 486 | 4132 | 4282 | 33316 | 76528 |
| 315.148 | 119 | 261741 | 92912 | 198572 | 137778 |
| 315.194 | 532 | 25760 | 50490 | 16410 | 40811 |
| 316.137 | 230 | 11311 | 20536 | 39783 | 80931 |
| 317.054 | 521 | 1584662 | 732225 | 1088260 | 1755566 |
| 320.869 | 94 | 134095 | 51487 | 110354 | 51865 |
| 322.189 | 140 | 5343 | 12593 | 53620 | 124141 |
| 322.189 | 460 | 8494 | 22523 | 89294 | 175649 |
| 325.209 | 473 | 98121 | 88881 | 200846 | 186266 |
| 327.078 | 444 | 26522 | 36328 | 18184 | 34240 |
| 328.137 | 131 | 125171 | 79373 | 84835 | 66928 |
| 328.192 | 156 | 2531 | 3146 | 31084 | 61084 |
| 328.192 | 480 | 4520 | 9654 | 42425 | 94511 |
| 330.751 | 142 | 19520 | 35331 | 6537 | 13577 |
| 334.157 | 132 | 102775 | 53878 | 68473 | 44217 |
| 339.266 | 421 | 74393 | 114440 | 50170 | 96818 |
| 341.192 | 187 | 2923 | 2492 | 61804 | 121676 |
| 341.192 | 472 | 8584 | 12350 | 71130 | 127437 |
| 344.012 | 526 | 274231 | 113614 | 201348 | 128731 |
| 344.030 | 106 | 14290 | 21407 | 12283 | 25029 |
| 344.184 | 143 | 3424 | 3078 | 38334 | 76429 |
| 344.184 | 518 | 10795 | 22443 | 35029 | 67010 |
| 345.029 | 358 | 193650 | 95069 | 289855 | 195090 |
| 346.007 | 529 | 5407663 | 2240680 | 3812961 | 2029622 |
| 347.168 | 450 | 181721 | 503883 | 54349 | 121827 |
| 353.103 | 152 | 3442 | 4395 | 34975 | 74192 |
| 356.068 | 133 | 15908 | 17054 | 33402 | 34952 |
| 358.280 | 412 | 289063 | 357365 | 240187 | 424506 |
| 365.086 | 98 | 174449 | 103399 | 127171 | 61088 |
| 368.070 | 113 | 60166 | 41803 | 37097 | 33662 |
| 371.055 | 154 | 27566 | 49125 | 5422 | 17424 |
| 371.313 | 139 | 203470 | 108702 | 177440 | 123382 |
| 371.313 | 502 | 223476 | 155284 | 157166 | 98817 |
| 378.106 | 118 | 153246 | 218115 | 94296 | 100371 |
| 380.253 | 366 | 476973 | 152088 | 416866 | 144084 |
| 381.984 | 528 | 1099845 | 409204 | 837641 | 531873 |
| 383.980 | 529 | 210682 | 94338 | 149987 | 126375 |
| 385.624 | 108 | 107387 | 47771 | 94589 | 50478 |
| 392.285 | 497 | 220849 | 174334 | 163447 | 120468 |
| 396.034 | 91 | 96522 | 57295 | 113296 | 56721 |
| 396.999 | 111 | 58045 | 71549 | 39838 | 49099 |
| 399.345 | 440 | 27615 | 66019 | 21449 | 37360 |
| 416.211 | 448 | 5761 | 11286 | 28583 | 67815 |
| 416.313 | 563 | 7097 | 12878 | 5236 | 11414 |
| 419.313 | 488 | 103604 | 95729 | 189074 | 159399 |
| 421.159 | 147 | 150513 | 100927 | 80321 | 87155 |
| 429.238 | 93 | 90375 | 71278 | 91107 | 50863 |
| 432.308 | 462 | 1410243 | 2039116 | 707797 | 499631 |
| 437.233 | 103 | 11689 | 21767 | 20073 | 40950 |
| 448.303 | 423 | 612536 | 979519 | 231263 | 230558 |
| 449.306 | 428 | 133849 | 260285 | 28949 | 43929 |
| 450.318 | 468 | 5799295 | 8661201 | 2650576 | 2336635 |
| 464.021 | 91 | 46148 | 36849 | 62014 | 44553 |
| 472.300 | 470 | 95397 | 214327 | 17686 | 38842 |
| 478.999 | 96 | 16825 | 20003 | 16078 | 24940 |
| 544.891 | 89 | 150783 | 167816 | 225570 | 159715 |
| 556.857 | 94 | 889247 | 911185 | 1192311 | 740707 |
| 560.867 | 93 | 217452 | 274603 | 303087 | 231014 |
| 566.885 | 94 | 1238433 | 1403486 | 1774593 | 1233950 |
| 567.890 | 93 | 79356 | 101188 | 113782 | 100629 |
| 577.845 | 91 | 20183 | 18078 | 30068 | 28631 |
| 582.860 | 94 | 814865 | 918952 | 1011766 | 802267 |
| 584.857 | 92 | 27696 | 35797 | 30021 | 35829 |
| 603.594 | 78 | 447419 | 654801 | 641188 | 631117 |
| 603.686 | 78 | 682471 | 1194639 | 1086805 | 2045193 |
| 603.866 | 80 | 445692 | 625655 | 621463 | 559189 |
| 603.960 | 78 | 279490 | 394311 | 410063 | 378751 |
| 634.798 | 94 | 27816 | 39926 | 21759 | 38669 |
| 650.846 | 92 | 232579 | 288269 | 223523 | 363504 |
| 656.792 | 93 | 94771 | 121693 | 65697 | 108927 |
| 663.955 | 80 | 1126316 | 2160325 | 2749845 | 5887454 |
| 664.155 | 81 | 547335 | 756691 | 807989 | 932981 |
| 666.820 | 93 | 81220 | 111717 | 77332 | 171955 |

**C. Using intensity values with log2 transformation of data, q= 0.1**

| ***m/z*** | **time (s)** | **Control Mean** | **Control SD** | **AMD Mean** | **AMD SD** |
| --- | --- | --- | --- | --- | --- |
| 101.416 | 121 | 505049 | 435687 | 593406 | 320168 |
| 102.066 | 112 | 50481 | 34125 | 82237 | 80967 |
| 134.044 | 473 | 212541 | 224627 | 153796 | 166281 |
| 146.049 | 156 | 307620 | 234300 | 183606 | 200307 |
| 148.093 | 153 | 190744 | 187213 | 165307 | 193881 |
| 157.083 | 114 | 323191 | 140249 | 235983 | 134922 |
| 174.562 | 105 | 24892 | 46771 | 12161 | 22907 |
| 182.080 | 459 | 230042 | 235241 | 250175 | 239878 |
| 186.221 | 329 | 2077 | 2717 | 91286 | 180950 |
| 196.060 | 362 | 32370 | 73119 | 126527 | 312913 |
| 219.085 | 386 | 128776 | 140593 | 65404 | 96988 |
| 219.097 | 400 | 59328 | 104409 | 19815 | 40126 |
| 220.556 | 108 | 43919 | 58887 | 19256 | 31652 |
| 222.056 | 329 | 14650 | 51239 | 167768 | 363194 |
| 229.140 | 477 | 77686 | 84239 | 113757 | 79703 |
| 239.090 | 145 | 431108 | 639378 | 190509 | 291712 |
| 243.037 | 106 | 88713 | 38269 | 189508 | 169598 |
| 244.189 | 147 | 832445 | 836177 | 78238 | 40736 |
| 245.193 | 145 | 90228 | 110175 | 1670 | 3129 |
| 255.231 | 526 | 7705 | 19010 | 7603 | 13424 |
| 256.109 | 114 | 141882 | 136719 | 164764 | 136841 |
| 283.003 | 118 | 25008 | 47784 | 32563 | 43680 |
| 285.070 | 252 | 38512 | 76227 | 30031 | 64205 |
| 288.137 | 129 | 35524 | 31246 | 20516 | 34339 |
| 293.586 | 108 | 140472 | 73948 | 104440 | 58968 |
| 294.153 | 115 | 85793 | 53404 | 67953 | 71885 |
| 295.112 | 128 | 35554 | 25603 | 27979 | 43178 |
| 310.187 | 141 | 3413 | 3116 | 28385 | 54808 |
| 315.148 | 119 | 261741 | 92912 | 198572 | 137778 |
| 315.194 | 532 | 25760 | 50490 | 16410 | 40811 |
| 316.137 | 230 | 11311 | 20536 | 39783 | 80931 |
| 317.054 | 521 | 1584662 | 732225 | 1088260 | 1755566 |
| 322.189 | 460 | 8494 | 22523 | 89294 | 175649 |
| 325.209 | 473 | 98121 | 88881 | 200846 | 186266 |
| 327.078 | 444 | 26522 | 36328 | 18184 | 34240 |
| 328.137 | 131 | 125171 | 79373 | 84835 | 66928 |
| 328.192 | 156 | 2531 | 3146 | 31084 | 61084 |
| 328.192 | 480 | 4520 | 9654 | 42425 | 94511 |
| 330.751 | 142 | 19520 | 35331 | 6537 | 13577 |
| 341.192 | 187 | 2923 | 2492 | 61804 | 121676 |
| 341.192 | 472 | 8584 | 12350 | 71130 | 127437 |
| 344.184 | 143 | 3424 | 3078 | 38334 | 76429 |
| 346.007 | 529 | 5407663 | 2240680 | 3812961 | 2029622 |
| 347.168 | 450 | 181721 | 503883 | 54349 | 121827 |
| 353.103 | 152 | 3442 | 4395 | 34975 | 74192 |
| 368.070 | 113 | 60166 | 41803 | 37097 | 33662 |
| 371.055 | 154 | 27566 | 49125 | 5422 | 17424 |
| 371.313 | 139 | 203470 | 108702 | 177440 | 123382 |
| 378.106 | 118 | 153246 | 218115 | 94296 | 100371 |
| 381.984 | 528 | 1099845 | 409204 | 837641 | 531873 |
| 383.980 | 529 | 210682 | 94338 | 149987 | 126375 |
| 385.624 | 108 | 107387 | 47771 | 94589 | 50478 |
| 396.034 | 91 | 96522 | 57295 | 113296 | 56721 |
| 416.211 | 448 | 5761 | 11286 | 28583 | 67815 |
| 429.238 | 93 | 90375 | 71278 | 91107 | 50863 |
| 432.308 | 462 | 1410243 | 2039116 | 707797 | 499631 |
| 448.303 | 423 | 612536 | 979519 | 231263 | 230558 |
| 449.306 | 428 | 133849 | 260285 | 28949 | 43929 |
| 450.318 | 468 | 5799295 | 8661201 | 2650576 | 2336635 |
| 464.021 | 91 | 46148 | 36849 | 62014 | 44553 |
| 478.999 | 96 | 16825 | 20003 | 16078 | 24940 |
| 560.867 | 93 | 217452 | 274603 | 303087 | 231014 |
| 566.885 | 94 | 1238433 | 1403486 | 1774593 | 1233950 |
| 567.890 | 93 | 79356 | 101188 | 113782 | 100629 |
| 582.860 | 94 | 814865 | 918952 | 1011766 | 802267 |
| 603.594 | 78 | 447419 | 654801 | 641188 | 631117 |
| 603.686 | 78 | 682471 | 1194639 | 1086805 | 2045193 |
| 603.866 | 80 | 445692 | 625655 | 621463 | 559189 |
| 603.960 | 78 | 279490 | 394311 | 410063 | 378751 |
| 650.846 | 92 | 232579 | 288269 | 223523 | 363504 |
| 656.792 | 93 | 94771 | 121693 | 65697 | 108927 |
| 663.955 | 80 | 1126316 | 2160325 | 2749845 | 5887454 |
| 664.155 | 81 | 547335 | 756691 | 807989 | 932981 |
| 666.820 | 93 | 81220 | 111717 | 77332 | 171955 |

**D. Using intensity values with log2 transformation of data, q= 0.05**

| ***m/z*** | **time (s)** | **Control Mean** | **Control SD** | **AMD Mean** | **AMD SD** |
| --- | --- | --- | --- | --- | --- |
| 157.083 | 114 | 323191 | 140249 | 235983 | 134922 |
| 182.080 | 459 | 230042 | 235241 | 250175 | 239878 |
| 186.221 | 329 | 2077 | 2717 | 91286 | 180950 |
| 219.097 | 400 | 59328 | 104409 | 19815 | 40126 |
| 220.556 | 108 | 43919 | 58887 | 19256 | 31652 |
| 222.056 | 329 | 14650 | 51239 | 167768 | 363194 |
| 239.090 | 145 | 431108 | 639378 | 190509 | 291712 |
| 243.037 | 106 | 88713 | 38269 | 189508 | 169598 |
| 244.189 | 147 | 832445 | 836177 | 78238 | 40736 |
| 245.193 | 145 | 90228 | 110175 | 1670 | 3129 |
| 256.109 | 114 | 141882 | 136719 | 164764 | 136841 |
| 283.003 | 118 | 25008 | 47784 | 32563 | 43680 |
| 288.137 | 129 | 35524 | 31246 | 20516 | 34339 |
| 294.153 | 115 | 85793 | 53404 | 67953 | 71885 |
| 295.112 | 128 | 35554 | 25603 | 27979 | 43178 |
| 315.148 | 119 | 261741 | 92912 | 198572 | 137778 |
| 316.137 | 230 | 11311 | 20536 | 39783 | 80931 |
| 322.189 | 460 | 8494 | 22523 | 89294 | 175649 |
| 327.078 | 444 | 26522 | 36328 | 18184 | 34240 |
| 328.137 | 131 | 125171 | 79373 | 84835 | 66928 |
| 330.751 | 142 | 19520 | 35331 | 6537 | 13577 |
| 341.192 | 472 | 8584 | 12350 | 71130 | 127437 |
| 347.168 | 450 | 181721 | 503883 | 54349 | 121827 |
| 353.103 | 152 | 3442 | 4395 | 34975 | 74192 |
| 371.055 | 154 | 27566 | 49125 | 5422 | 17424 |
| 371.313 | 139 | 203470 | 108702 | 177440 | 123382 |
| 385.624 | 108 | 107387 | 47771 | 94589 | 50478 |
| 396.034 | 91 | 96522 | 57295 | 113296 | 56721 |
| 432.308 | 462 | 1410243 | 2039116 | 707797 | 499631 |
| 449.306 | 428 | 133849 | 260285 | 28949 | 43929 |
| 464.021 | 91 | 46148 | 36849 | 62014 | 44553 |
| 566.885 | 94 | 1238433 | 1403486 | 1774593 | 1233950 |
| 603.594 | 78 | 447419 | 654801 | 641188 | 631117 |
| 603.686 | 78 | 682471 | 1194639 | 1086805 | 2045193 |
| 603.866 | 80 | 445692 | 625655 | 621463 | 559189 |
| 650.846 | 92 | 232579 | 288269 | 223523 | 363504 |
| 656.792 | 93 | 94771 | 121693 | 65697 | 108927 |
| 664.155 | 81 | 547335 | 756691 | 807989 | 932981 |
| 666.820 | 93 | 81220 | 111717 | 77332 | 171955 |
