# Supplementary material for: Metabolome-Wide Association Study of Neovascular Age-Related Macular Degeneration
Source: PLoS One. 2013 Aug 27;8(8):e72737. doi: 10.1371/journal.pone.0072737 (PMC3754980; doi:10.1371/journal.pone.0072737)
Supplement: Table S2 — (DOCX) [file pone.0072737.s005.docx]

**Table S2.** Intersection of the 94 features that differed between controls and NVAMD patients by FDR of raw data at q= 0.05 with the 132 features that differed by FDR of log2 transformed data at *q*= 0.2.

| ***m/z*** | **RT (s)** |
| --- | --- |
| 144.101 | 103 |
| 157.083 | 114 |
| 186.221 | 329 |
| 208.096 | 340 |
| 219.085 | 386 |
| 220.556 | 108 |
| 222.112 | 449 |
| 244.189 | 147 |
| 245.193 | 145 |
| 266.171 | 144 |
| 278.039 | 113 |
| 292.152 | 400 |
| 293.586 | 108 |
| 310.187 | 141 |
| 320.869 | 94 |
| 322.189 | 140 |
| 325.209 | 473 |
| 328.137 | 131 |
| 328.192 | 156 |
| 334.157 | 132 |
| 341.192 | 187 |
| 341.192 | 472 |
| 344.012 | 526 |
| 344.184 | 143 |
| 345.029 | 358 |
| 346.007 | 529 |
| 353.103 | 152 |
| 356.068 | 133 |
| 365.086 | 98 |
| 368.070 | 113 |
| 371.055 | 154 |
| 381.984 | 528 |
| 416.211 | 448 |
| 419.313 | 488 |
| 421.159 | 147 |
| 448.303 | 423 |
| 449.306 | 428 |
| 450.318 | 468 |
| 472.300 | 470 |
| 656.792 | 93 |

Thirteen intersecting features for FDR of log2 transformed data at *q*= 0.05 are shown in red.
