# Supplementary material for: Metabolome-Wide Association Study of Neovascular Age-Related Macular Degeneration
Source: PLoS One. 2013 Aug 27;8(8):e72737. doi: 10.1371/journal.pone.0072737 (PMC3754980; doi:10.1371/journal.pone.0072737)
Supplement: Table S3 — (DOCX) [file pone.0072737.s006.docx]

**Table S3A.** Analysis of predictive accuracy (%) by support vector machine (SVM) based on 10-fold and Leave-one-out cross validation (LOOCV) evaluation of significant features obtained using raw (non-transformed) high-resolution metabolomics data.

|  | q<0.05; using raw intensities | q<0.2; using  log2 intensities | q<0.1; using log2 intensities | q<0.05; using log2 intensities |
| --- | --- | --- | --- | --- |
| Gaussian kernel;10-fold CV | 91.11 | 84.44 | 80.00 | 82.22 |
| Gaussian kernel; LOOCV | 91.11 | 86.67 | 86.67 | 86.67 |
| Polynomial kernel; 10fold CV | 62.22 | 55.56 | 57.78 | 60.00 |
| Polynomial kernel; LOOCV | 64.44 | 57.78 | 57.78 | 60.00 |
| Linear kernel; 10-fold CV | 95.56 | 91.11 | 86.67 | 86.67 |
| Linear kernel; LOOCV | 95.56 | 86.67 | 88.89 | 84.44 |

**Table S3B.** Analysis of predictive accuracy (%) by SVM based on 10-fold and LOOCV evaluation of significant features obtained using log2 transformed data.

|  | q<0.05; using raw intensities | q<0.2; using  log2 intensities | q<0.1; using log2 intensities | q<0.05; using log2 intensities |
| --- | --- | --- | --- | --- |
| Gaussian kernel;10-fold CV | 84.44 | 91.11 | 88.89 | 84.44 |
| Gaussian kernel; LOOCV | 86.67 | 93.33 | 93.33 | 86.67 |
| Polynomial kernel; 10fold CV | 71.11 | 60.00 | 62.22 | 75.56 |
| Polynomial kernel; LOOCV | 71.11 | 57.78 | 60.00 | 75.56 |
| Linear kernel; 10-fold CV | 95.56 | 88.89 | 93.33 | 95.56 |
| Linear kernel; LOOCV | 95.56 | 88.89 | 93.33 | 95.56 |
