# Supplementary material for: Metabolome-Wide Association Study of Neovascular Age-Related Macular Degeneration
Source: PLoS One. 2013 Aug 27;8(8):e72737. doi: 10.1371/journal.pone.0072737 (PMC3754980; doi:10.1371/journal.pone.0072737)
Supplement: Table S4 — (DOCX) [file pone.0072737.s007.docx]

**Table S4. Pathways associated with the non-transformed 94 FDR features at (q=0.05) identified using the MetScape plugin in Cytoscape.**

| **Pathways** |
| --- |
| Bile acid biosynthesis |
| Biopterin metabolism |
| Endohydrolysis of 1,4-alpha-D-glucosidic linkages in polysaccharides by alpha-amylase |
| Fructose and mannose metabolism |
| Galactose metabolism |
| Glycolysis and Gluconeogenesis |
| Glycosphingolipid biosynthesis - ganglioseries |
| Glycosphingolipid metabolism |
| Lysine metabolism |
| O-Glycan biosynthesis |
| Pentose phosphate pathway |
| Phosphatidylinositol phosphate metabolism |
| Pyrimidine metabolism |
| Tyrosine metabolism |
| Urea cycle and metabolism of arginine, proline, glutamate, aspartate and asparagine |
| Vitamin B1 (thiamin) metabolism |
| Vitamin B5 - CoA biosynthesis from pantothenate |
